# Association between S-ketamine induced changes in glutamate levels in the pregenual anterior cingulate cortex and plasma brain-derived neurotrophic factor in healthy subjects

**DOI:** 10.3389/fpsyt.2025.1662051

**Published:** 2025-10-31

**Authors:** Leonard Marx, Zümrüt Duygu Sen, Lena Vera Danyeli, Meng Li, Tanja Brigadski, Volkmar Leßmann, Martin Walter

**Affiliations:** ^1^ Department of Psychiatry and Psychotherapy, Jena University Hospital, Jena, Germany; ^2^ Center for Intervention and Research on Adaptive and Maladaptive Brain Circuits Underlying Mental Health (C-I-R-C), Jena, Germany; ^3^ German Center for Mental Health (DZPG), Jena, Germany; ^4^ Institute of Physiology, Otto-von-Guericke-University, Magdeburg, Germany; ^5^ Department of Informatics and Microsystems Technology, University of Applied Sciences Kaiserslautern, Zweibrücken, Germany; ^6^ Center for Intervention and Research on Adaptive and Maladaptive Brain Circuits Underlying Mental Health (C-I-R-C), Magdeburg, Germany; ^7^ German Center for Mental Health (DZPG), Magdeburg, Germany

**Keywords:** ketamine, glutamate, brain-derived neurotrophic factor, magnetic resonance spectroscopy, anterior cingulate cortex, depression

## Abstract

**Introduction:**

Ketamine’s antidepressant effects have been linked to its modulation of glutamatergic neurotransmission and synaptic plasticity. However, the precise roles of both glutamate (Glu) levels and brain-derived neurotrophic factor (BDNF) in this process remain incompletely understood.

**Methods:**

This study examined the relationship between ketamine-induced changes in Glu levels and peripheral BDNF levels using data from a randomized, placebo-controlled crossover design. Proton magnetic resonance spectroscopy (7 Tesla ^1^H-MRS) assessing Glu concentrations in the pregenual anterior cingulate cortex (pgACC) and plasma BDNF levels were measured one hour before and 24 hours after either S-ketamine or placebo infusions in 35 healthy male subjects.

**Results:**

Linear regression analysis revealed a significant interaction between treatment condition and relative changes in Glu on BDNF level changes, with a trend-level positive correlation between changes in Glu and BDNF levels observed only in the ketamine group.

**Discussion:**

These findings provide initial *in vivo* support for the hypothesis that ketamine’s effects on BDNF dynamics are linked to its glutamatergic action.

## Introduction

Major depressive disorder (MDD) is a prevalent and debilitating psychiatric condition that affects approximately 300 million people globally, accounting for 4.3% of the global disease burden (1). Among those individuals suffering from MDD, about 30% do not respond adequately to standard antidepressant therapies and are therefore diagnosed with treatment resistant depression (TRD) (2, 3), underscoring the necessity for novel therapeutic interventions. Among these new treatment approaches are so-called rapid-acting antidepressants, with ketamine being the most extensively studied agent.

As a non-competitive N-methyl-D-aspartate (NMDA) receptor antagonist ketamine has demonstrated rapid antidepressant effects, attributed primarily to its acute influence on glutamatergic neurotransmission (4–6). Current models propose that ketamine administration induces a transient surge in extracellular Glu, which subsequently activates α-amino-3-hydroxy-5-methyl-4-isoxazolepropionic acid (AMPA) receptors (7). This activation is believed to initiate intracellular signaling pathways, including mechanistic target of rapamycin (mTOR) activation, resulting in enhanced synaptic plasticity and increased release of brain-derived neurotrophic factor (BDNF) (8–11).

The proposed glutamatergic surge observed in rodents is supported by experimental evidence in human subjects. Thus, increased concentration of glutamatergic metabolites in the prefrontal cortex following ketamine administration were reported in ¹H-MRS studies (12–15). However, some studies reported no systematically replicated effect (16) or even a decrease in Glu metabolism in both rodents (17) and the ventromedial prefrontal cortex (vmPFC) in humans, which partially includes the pregenual anterior cingulate cortex (pgACC) (18). These inconsistencies suggest considerable variation of effects across individuals. Variable glutamatergic effects have been related to different subjective baseline conditions, suggesting that covariance of ketamine-induced effects may shed light on the relationship of the different affected processes (19).

This also highlights the importance of the pgACC as a central node within the default mode network (DMN), critically involved in the pathophysiology of depression and serving as a key mediator of ketamine’s antidepressant effects (20). In fact, converging evidence suggests that Glu concentrations are reduced within this region in depressed patients (21, 22) and restored pgACC activity has been reported as a result of successful antidepressant treatment (23). Interestingly, pgACC activity was shown to correlate with Glu increase 24 hours after ketamine infusion (15). Also, Danyeli et al. reported that the ketamine-induced immediate changes in DMN functional connectivity was associated with Glu level increase in pgACC 24 hours after the infusion (13), further supporting a critical role of the pgACC as a target region for investigating ketamine-induced neurochemical changes.

In addition to glutamatergic mechanisms, the neuronal growth factor BDNF is involved in MDD pathology and contributes to ketamine’s antidepressant effects. While it is widely acknowledged that depressive symptoms negatively correlate with BDNF concentrations (24, 25) whereas successful antidepressant treatment typically induces elevated BDNF levels (26), findings regarding peripheral BDNF after ketamine administration remain inconsistent: Some studies report increased peripheral BDNF levels in response to ketamine infusion, particularly in treatment responders (27, 28), while others detect no significant BDNF changes (29–31). Interestingly, previous studies have shown that the BDNF genotype plays a mediating role in this context as the common BDNF single nucleotide polymorphism leading to an amino acid substitution (valine to methionine) at codon 66 (val66met) within the 5’ pro-BDNF sequence is associated with impaired intracellular packaging and reduced activity-dependent secretion of BDNF (32–34).

To address the inconsistencies regarding ketamine’s influence on central brain Glu and peripheral BDNF blood levels, this secondary analysis of data from a randomized, placebo-controlled, double-blind mechanistic crossover study in healthy individuals aimed to examine whether ketamine-induced changes in Glu and BDNF levels are associated. In line with previous investigations of ketamine effects on BDNF (28) and Glu (35) in healthy subjects, the careful selection of healthy participants in this study minimized potential confounding factors such as antidepressant medication, disease duration or comorbidities, ensuring that the observed effects could be solely attributed to the experimental conditions. Enhanced insights into the mechanisms underlying ketamine’s rapid antidepressant effects could help to identify potential biomarkers for treatment response and facilitate the development of novel antidepressant treatments with improved safety profiles and fewer side effects.

## Materials and methods

### Study design

This study was conducted as a secondary analysis of data from a randomized, placebo-controlled, double-blind crossover study that investigated the immediate and delayed effects of a single sub-anesthetic S-ketamine infusion on functional connectivity and glutamatergic metabolite levels (13). The study enrolled 35 healthy male participants, aged between 18 and 35 years, with a mean age of 25.1 years (for complete demographic data, see **Supplementary Table 1**). The selection of male subjects in this mechanistic trial was based on previous observations of differential ketamine effects between males and females (36), which could have influenced the primary interindividual covariations of interest. Since the study did not aim to assess therapeutic outcomes, potential gender biases limiting generalizability to clinical use were not a primary concern. Subjects were carefully screened to exclude neurological or physical constraints, severe illnesses, and, according to the fourth edition of the Statistical Manual of Mental Disorders (DSM-IV) criteria (37), current or past psychiatric disorders as well as substance use disorders, through demographic questionnaires, clinical interviews (Structured Clinical Interview for DSM-IV, SCID) and physical examinations (38).

Participants underwent two treatment sessions, receiving either S-ketamine hydrochloride (0.33 mg/kg body weight; Ketanest^®^ S; Pfizer Pharma) or placebo (0.9% saline) administered via an infusion pump (Injectomat^®^ MC Agilia; Fresenius Kabi). The infusion protocol comprised an initial bolus (0.11 mg/kg body weight) delivered over 8 minutes, followed by a brief pause of 2 minutes, and subsequently a maintenance dose (0.22 mg/kg body weight) administered over 40 minutes. Blood samples for BDNF analysis were collected one hour before infusion and 24 hours after infusion completion, both prior to the respective MRI session. Sampling occurred within a daytime range between 08:15 and 19:36 across participants. Sessions were separated by a washout period of approximately three weeks to prevent carry-over effects (a schematic of the study protocol is provided in **Supplementary Figure S1**). All participants provided written informed consent. The study protocol was approved by the Otto-von-Guericke-University Magdeburg Institutional Review Board and conducted according to Declaration of Helsinki guidelines and local legal provisions.

### Magnetic resonance spectroscopy

As reported by Danyeli et al., participants underwent magnetic resonance spectroscopy (MRS) measurements on an ultra-high field 7 Tesla MR scanner (Siemens Healthineers, Erlangen, Germany) using a 32-channel head coil. Spectra were acquired from the pgACC using a stimulated-echo acquisition mode (STEAM) sequence with the following parameters: voxel size = 20 × 15 × 10 mm³, echo time (TE) = 20 ms, repetition time (TR) = 3000 ms, mixing time (TM) = 10 ms, bandwidth = 2800 Hz, and 128 averages. A single average water signal was recorded as an internal reference for quantification and eddy current correction. The voxel was placed individually for each participant according to anatomical landmarks, touching the genu of the corpus callosum while avoiding the callosomarginal artery, and tilted to align with the anterior commissure-posterior commissure plane. MRS data were acquired before and 24 hours after intravenous infusion of either S-ketamine (0.33 mg/kg body weight) or placebo (0.9% saline). Glu concentrations were quantified as absolute values using water as an internal reference. Data preprocessing and metabolite quantification were performed with LCModel (version 6.3.0), and spectra were visually inspected for quality assurance, excluding spectra with a Cramér-Rao lower bound (CRLB) >20%, line width >24 Hz, or signal-to-noise ratio <20 in accordance with previous investigations (39, 40). These thresholds account for the technical challenges of achieving optimal data quality in the pgACC region, which is located near air-tissue interfaces. Spectral quality assessment for the analyzed timepoints revealed acceptable data quality, with a mean linewidth (full width at half maximum) of 6.72 ± 2.59 Hz and a mean signal-to-noise ratio of 41.05 ± 5.97 across the included spectra. After excluding measurements that did not fulfil quality criteria, spectra for Glu analysis were available from 29 participants in the placebo condition at both baseline and 24 hours post-infusion, and from 32 participants in the S-ketamine condition. For further methodological details and voxel placement illustrations related to the MRS data, please refer to (13).

### Blood sampling

Two separate blood samples were collected at each timepoint. One in EDTA-anticoagulated tubes for BDNF genotyping (BD Vacutainer, K3E, 7.2 mg, REF 368860) and one in heparin-containing tubes for BDNF quantification (BD Vacutainer, LH, 68 I.U. REF 368884). Both samples were collected before and 24 hours after infusions using a BD Vacutainer Safety-Lok Blood Collection Set, with participants seated.

Heparin tubes were immediately placed on ice and centrifuged within 15 minutes at 20°C and 2000 relative centrifugal force (RcF) for 15 minutes. The resulting plasma supernatants were stored at -80°C until further analysis. Plasma BDNF levels were quantified using a sandwich enzyme-linked immunosorbent assay (ELISA) according to the manufacturer’s instructions (DuoSet ELISA Development Kit, R&D Systems, Wiesbaden, Germany). Photometric analysis of samples was performed with an ELISA reader (Infinite^®^ 200, Tecan, Switzerland). For quantification of BDNF, plasma samples were diluted 1:8 and serum samples 1:128. Dilution linearity was obtained in the range of 1:6, 1:8, 1:10 and 1:12 for plasma samples (coefficient of variation: minimum–maximum, 2.2–5.8) and in the range of 1:64, 1:80, 1:100, 1:128, 1:150 and 1:180 for serum samples [coefficient of variation: minimum–maximum, 0.6–4.8; see (41)]. This methodology is consistent with current best practice recommendations for BDNF quantification in plasma samples (41–43).

The BDNF genotype of each subject was assessed using polymerase chain reaction (PCR) followed by restriction fragment length polymorphism (RFLP) analysis to investigate a potential influence of the val66met polymorphism (NCBI accession number: rs6265) on BDNF blood levels after ketamine administration, with forward primer 5′-GCATCCCGGTGAAAGAAAGCCCTAAC-3′ and reverse primer 5′-GCCCCTGCAGCCTTCTTTTGTGTAAC-3′ for amplification. PCR was performed using MyTaq™ DNA Polymerase (Bioline, Meridian Bioscience), and the resulting amplicon was digested with Eco72I FastDigest enzyme (Thermo Scientific). The resulting fragments were analyzed by electrophoresis on a 1% agarose gel stained with MIDORI Green Advance (Nippon Genetics Europe). Assays were conducted under blinded conditions. Missing BDNF blood level data occurred in 32 out of 210 samples. Of these, 31 values fell below the assay’s limit of quantification (LOQ = 23.4 pg/mL), and one additional value was missing due to technical issues during blood withdrawal. Relative BDNF changes could thus ultimately be calculated for 27 participants receiving S-ketamine and 24 in placebo controls. Therefore, the final analyzed correlation pairs between relative changes in Glu and BDNF were *n* = 20 in the placebo condition and *n* = 26 in the S-ketamine condition.

### Statistical analysis

Given the consistently observed circadian variation of plasma BDNF levels (44–46), values were statistically adjusted for the exact blood sampling time. Specifically, residuals were obtained from a linear regression model with plasma BDNF levels predicted by numerically scaled and rescaled blood collection times. To approximate a normal distribution suitable for subsequent statistical analyses, these residuals were shifted by adding the absolute minimum residual value (to eliminate negative numbers) i.e., plus one, and were then log-transformed. This methodological approach aligns with established procedures previously applied in related studies examining plasma BDNF levels (28). Glu concentrations were normalized to the combined gray matter (GM) and white matter (WM) volume within each pgACC voxel. For this correction, GM and WM fractions were extracted from individual average T1-weighted reference images using CAT12 in SPM12 (http://www.neuro.uni-jena.de/cat/), bias-field corrected and segmented into GM, WM and cerebrospinal fluid (CSF), with spatial normalization performed via ANTs (ANTs 2.2.0). The resulting GM+WM fractions were then used to scale fitted metabolite levels, as detailed in (13).

Relative changes for both metabolites were then calculated as the difference between measurements taken 24 hours post-infusion and baseline values, divided by the baseline values [(24h post-infusion - baseline)/baseline]. Linear regression analysis was conducted to evaluate the relationship between relative changes in BDNF and relative changes in Glu concentrations across the two treatment conditions. The regression model included treatment (S-ketamine *vs* placebo), relative Glu changes, and their interaction term as predictors for the relative BDNF changes. Normality of residuals was assessed visually using histograms and Q-Q plots and statistically via Shapiro test (W = 0.95946, p = 0.1092), indicating no significant deviation from normality with the overall model reaching statistical significance (F(3,42) = 2.832, p = 0.0497). Due to the non-normal distribution of relative Glu changes in the S-ketamine group, a *post-hoc* Spearman’s rank correlation was performed to investigate the relationship between relative changes in Glu and BDNF separately for each treatment condition. Bonferroni correction was applied to account for multiple dependent variables, resulting in adjusted significance levels of α = 0.0335 for the S-ketamine correlation (n = 26) and α = 0.025 for the Placebo correlation (n = 20). The adjusted significance thresholds were calculated using the Bonferroni adjustment tool provided at www.quantitativeskills.com, with different adjusted alpha levels reflecting the unequal number of comparisons or dependent variables tested within each treatment condition.

Following the approach described by Danyeli et al., who examined the relationship between delayed Glu levels and resting state functional connectivity changes with respect to their baseline values (13), we applied the same analytical method to our BDNF measurements. Specifically, correlations were calculated using Oldham’s method, assessing the relationship between the change from baseline (24 hours post-infusion minus baseline) and the mean of these two time points (24 hours post-infusion plus baseline divided by two) (47) in order to detect potential systematic biases or proportional changes. In addition, paired *t* tests were conducted separately for BDNF and Glu concentrations to examine differences between baseline and 24 hours after infusion measurements within each treatment condition (S-ketamine and placebo), as well as to assess differences between the two treatment conditions at each respective time point.

## Results

### Delayed effects of S-ketamine on pgACC glutamatergic metabolites

As the present study builds on MRS findings from a previous investigation by our group, key results from that work are briefly summarized here to provide context; for full statistical details, see (13). Danyeli et al. reported a significant increase in Glu concentrations in the pgACC 24 hours after S-ketamine infusion, while no such effect was observed in the placebo group. Furthermore, individuals with lower initial Glu levels showed greater post-infusion increases, suggesting a baseline-dependent modulation. Given that no significant changes in glutamine (Gln), Glx (combined Glu and Gln), or the Gln/Glu ratio were found in either treatment arm, no additional analyses of these metabolites were conducted in the present study.

### Delayed effects of S-ketamine on plasma BDNF levels

To ensure consistency with our main analysis, plasma BDNF analyses were restricted to participants with available data for both Glu and BDNF. No significant differences in plasma BDNF levels were observed between the ketamine and placebo condition at baseline (t(27) = −1.01, p = 0.321) or 24 hours post-infusion (t(20) = 0.47, p = 0.644). Interestingly, within-group analyses revealed a significant decrease in BDNF from baseline to 24 hours post-infusion in the ketamine group (t(25) = 2.39, α = 0.025, p = 0.0249), while BDNF levels in the placebo group did not change significantly between the two timepoints (t(19) = 1.12, α = 0.025, p = 0.278; for details, see **Supplementary Figures S2A, B**). Furthermore, no significant correlation was found between the BDNF level change and the respective baseline BDNF level (rs = 0.186, α = 0.05, p = 0.195), suggesting that, unlike Glu, Plasma BDNF levels do not exhibit a baseline-dependent modulation following S-ketamine administration. For the exact sample size of each genotype group, see **Supplementary Table 1**.

### Association of delayed effects of S-ketamine on pgACC Glu and plasma BDNF levels

**Figure 1:**
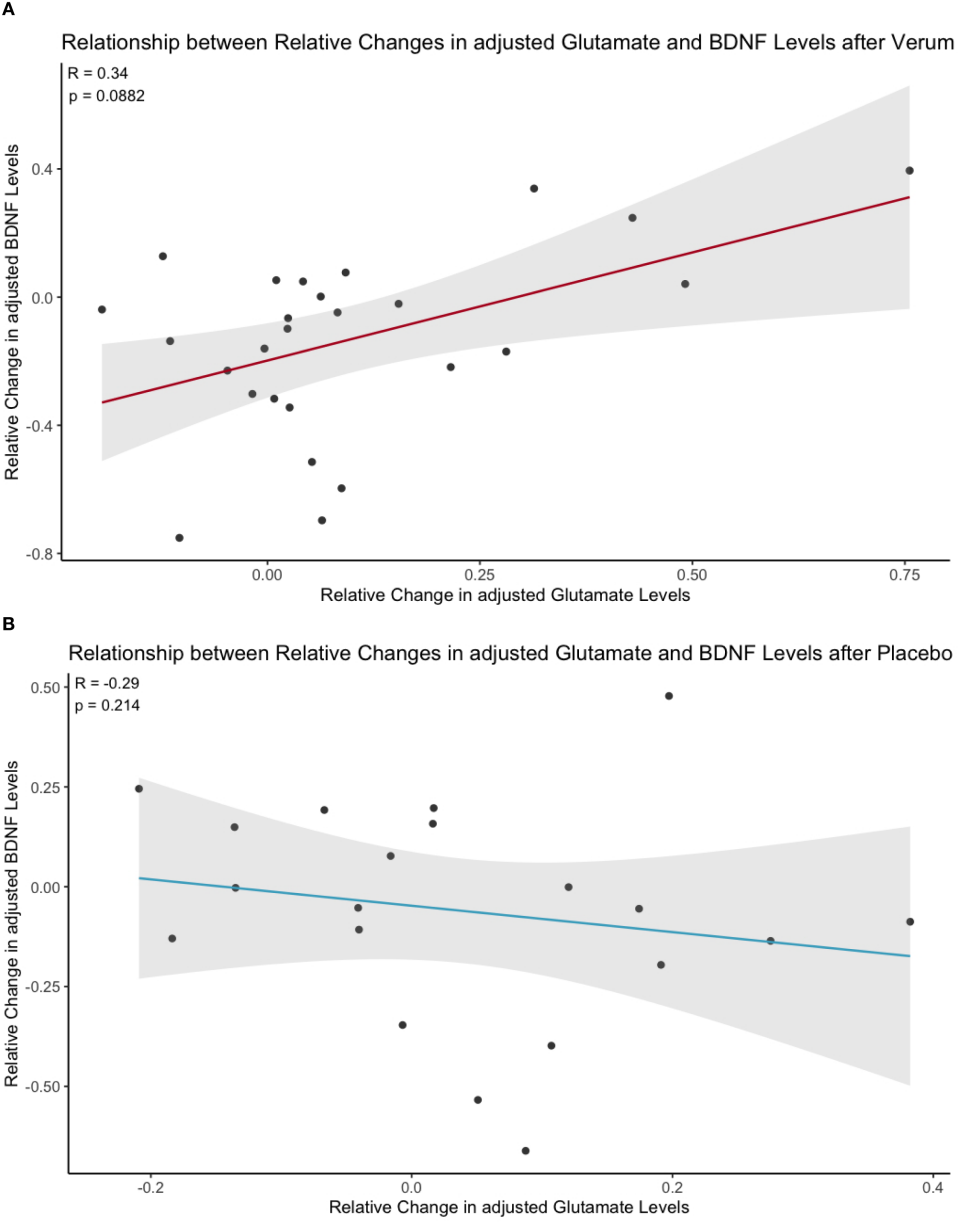
Scatter plot of the relationship between relative changes in Glu (adjusted for voxel gray and white matter content) and plasma BDNF (adjusted for sampling time) 24 hours after S-ketamine **(A)** or placebo **(B)** infusion. Regression lines indicate a trend-level positive correlation for S-ketamine (Spearman’s rho = 0.34, p = 0.088, adjusted α = 0.0335) but not for placebo (Spearman’s rho = -0.29, p = 0.2138, adjusted α = 0.025); shaded area represents the 95% confidence interval.

Linear regression analysis revealed a significant interaction effect between relative Glu changes and treatment condition (β = 1.00482, SE = 0.46990, t = 2.138, p = 0.0383) on relative BDNF changes (for detailed model parameters, see **Table 1**). Thus, the relationship between Glu and BDNF changes significantly differed between the two treatment groups. Spearman correlation analyses showed a trend-level positive correlation (rs = 0.34, p = 0.088, adjusted α = 0.0335) in the S-ketamine condition (**Figure 1A**; n = 26), whereas a non-significant negative correlation was detected (rs = -0.29, p = 0.2138, adjusted α = 0.025) in the placebo condition (**Figure 1B**; n = 20). Neither relative Glu changes alone (β = -0.33009, p = 0.4081) nor treatment condition alone (β = -0.15023, p = 0.0837) showed a statistically significant main effect.

**Table 1:** Results of the linear regression analysis assessing the interaction between relative Glu changes and treatment condition (S-ketamine *vs*. placebo) on relative plasma BDNF changes.

Predictor	*⁠β*	SE	*t*	*p*
(Intercept)	-0.048	0.062	-0.077	0.444
Relative Glu Change	-0.330	0.040	-0.836	0.408
Treatment (S-ketamine)	-0.150	0.085	-1.772	0.084.
Relative Glu Change: Treatment (S-ketamine)	1.004	0.470	2.138	0.038*

Glu, Glutamate; SE, Standard Error.

‘*’ p < 0.05, ‘.’ p < 0.1.

Further analyses were performed to investigate potential influences of Age, Body Mass Index (BMI), and Genotype (categorized as Val/Val and Val/Met, with only two subjects exhibiting the Met/Met genotype) on this relationship. When each of these covariates was included separately, neither Age (β =-0.00013, p = 0.9888), BMI (β = 0.00015, p = 0.9926), nor Genotype showed significant associations with the dependent variable. Specifically, with Val/Val as the reference category (selected due to its larger sample size, n = 24, providing more stable parameter estimates), neither Met/Met (β = -0.02194, p = 0.8967) nor Val/Met genotypes (β = -0.06347, p = 0.5138) demonstrated significant effects on BDNF relative change. Importantly, the significant interaction between relative Glu changes and treatment on BDNF level changes remained robust after controlling for each of these covariates individually (p-values remained <0.05), confirming that this interaction effect was independent of age, BMI, and genotype (full covariate models are reported in **Supplementary Tables 2–4**).

## Discussion

In this study, we identified an association between ketamine-induced changes in pgACC glutamate and peripheral plasma BDNF levels following S-ketamine administration. BDNF levels displayed a significant decrease 24h after infusion in the S-ketamine arm. Changes in Glu concentrations have been described comprehensively in a prior report by Danyeli et al. (13). Notably, despite this general decrease in BDNF, individuals demonstrating larger Glu elevations tended to show smaller reductions or even increases in BDNF levels, indicating a potential coupling between neurotrophic response and central Glu metabolism. While previously reported findings showed a baseline-dependent increase in Glu 24 hours after S-ketamine infusion (13), no such modulation was observed for BDNF concentrations in the present study.

Taken together, these observations support a model in which ketamine’s rapid antidepressant action emerges from an initial Glu surge – likely pronounced in individuals with low baseline Glu (13) – triggering BDNF-mediated neuroplastic changes. This glutamatergic–neurotrophic cascade hypothesis is supported by pharmacological evidence on ketamine’s signaling pathway stating that BDNF upregulation is in multiple ways dependent on glutamatergic activation. Namely, preclinical studies have shown that ketamine enhances BDNF–TrkB signaling by relieving translational repression through inhibition of eukaryotic elongation factor 2 kinase (eEF2K), by facilitating TrkB receptor activation, and by engaging downstream signaling pathways such as phosphoinositide 3-kinase–protein kinase B (PI3K–Akt) and mitogen-activated protein kinase–extracellular signal-regulated kinase (MEK–ERK) (48, 49). Furthermore, ketamine-induced glutamatergic transmission may trigger BDNF release on a timescale faster than BDNF translation (10, 11, 50). This glutamatergic–neurotrophic cascade hypothesis is in accordance with the multistage model by Walter et al. arguing that ketamine first induces an acute glutamatergic disinhibition, followed by neuroplastic changes that enable a sustained antidepressant effect (21). Although our findings suggest a metabolic coupling after ketamine treatment, the proposed sequence requires further validation through studies incorporating multiple post-infusion time points and clinical populations.

Whilst it is important to note that the proposed central mechanisms of ketamine action and the assumption that peripheral BDNF levels reflect central concentrations are largely supported by rodent models and *in vitro* studies (51), plasma BDNF has also been shown to correlate with CSF BDNF concentrations in humans (52). Moreover, decreased BDNF levels in hippocampal and prefrontal cortex tissue as well as in CSF have been reported in depressed patients (53) with antidepressant treatment typically inducing elevations in peripheral levels of the neurotrophic factor (26). Although these findings collectively support the validity of using peripheral BDNF as a proxy for central neurotrophic activity, it should be further acknowledged that findings regarding the correlation between peripheral serum and plasma BDNF remain inconsistent (54, 55). In the current study, only plasma BDNF concentrations were measured. As platelets store a significant amount of BDNF in the blood stream and are known to release it upon clotting (56), plasma BDNF levels are significantly lower than in serum, but potentially better reflect BDNF brain levels since platelets do not cross the blood-brain barrier (57). Moreover, MDD has been shown to induce platelet alterations and antidepressants as well as antiaggregating medications influence BDNF release from platelets (58). Given that serum and plasma BDNF measurements are not interchangeable, future studies are needed to verify whether the observed association with Glu also holds up in serum measurements. Due to the generally very low concentrations of BDNF in plasma, it remains challenging to accurately quantify plasma BDNF levels. This was also evident in our study, where a moderate proportion of measurements were left-censored: 31 out of 32 missing values fell below the assay’s lower limit of quantification (LOQ), while only one value was missing due to technical issues during blood withdrawal. However, because relative changes were calculated and missing data were handled systematically, this limitation is unlikely to have introduced significant bias.

Further limitations should be considered. Firstly, the study was restricted to male participants, which limits the generalizability of the findings regarding sex differences in BDNF dynamics. This restriction reflects the fact that the current analysis was part of a larger investigation examining the relationship between the immediate and delayed neural effects of S-ketamine, for which relatively stable intersubject variability in target parameters was essential. Secondly, emerging evidence suggests that (R)-ketamine may rely more heavily on BDNF/TrkB signaling compared to (S)-ketamine (59, 60). These indications suggest that the relative contributions of Glu-dependent neuroplasticity and BDNF signaling may differ between the two isomers, highlighting the need for further research to elucidate their distinct pharmacological profiles.

Lastly, our findings demonstrate a Glu-BDNF association after ketamine administration only in healthy participants. Thus, future research should explore this correlation also in patient populations, thereby analyzing whether baseline Glu-BDNF coupling or its post-treatment modulation can predict clinical outcomes. If validated in larger clinical samples, this relationship may contribute to precision medicine approaches, aiding in the stratification of patients who are most likely to benefit from ketamine treatment and potentially guiding the development of novel interventions targeting the Glu-BDNF axis.

## Conclusion

The observed correlation between ketamine-induced glutamatergic and BDNF responses in this study suggests a meaningful functional link between these pathways in humans, previously well-documented only in animal models. Given the inconsistent findings on both BDNF and Glu levels across studies, interpreting peripheral BDNF as a standalone biomarker risks oversimplifying its role and may obscure key mechanistic insights in antidepressant research. Instead, our results emphasize the importance of assessing BDNF and Glu in tandem, as their combined analysis could yield a more integrated understanding of ketamine’s neurobiological effects and accelerate the identification of novel treatment targets.

## Data Availability

The original contributions presented in the study are included in the article/**Supplementary Material**. Further inquiries can be directed to the corresponding author.
