# Supplementary material for: Association between S-ketamine induced changes in glutamate levels in the pregenual anterior cingulate cortex and plasma brain-derived neurotrophic factor in healthy subjects
Source: Front Psychiatry. 2025 Oct 31;16:1662051. doi: 10.3389/fpsyt.2025.1662051 (PMC12616132; doi:10.3389/fpsyt.2025.1662051)
Supplement: Supplementary file 1 [file DataSheet1.docx]

**Supplementary Material**

**Authors**: Leonard Marx^1^, Zümrüt Duygu Sen^1,2,3^, Lena Vera Danyeli^1,2,3^, Meng Li^1,2^, Tanja Brigadski (https://orcid.org/0000-0001-9816-3964)^4,5^, Volkmar Leßmann (https://orcid.org/0000-0002-6863-323X)^2,3,4^, Martin Walter (https://orcid.org/0000-0001-7857-4483)^1,2,3^

**Correspondence**: Prof. Dr. Martin Walter: martin.walter@med.uni-jena.de; Department of Psychiatry and Psychotherapy, Jena University Hospital, Philosophenweg 3, 07743 Jena, Germany; Fax: +49 (3641) 9390102; Tel-Nr: +49 (3641) 9300

Supplementary Figure Legends

**Supplementary Fig. S1**

**Study design.** The randomized, placebo-controlled crossover study included two consecutive days per treatment arm. On day 1 (infusion day), participants underwent MR scanning, including MRS of the pregenual anterior cingulate cortex, before the infusion of S-ketamine (0.33 mg/kg) or placebo. Blood samples for BDNF measurement were collected 1 hour prior to infusion (baseline). On day 2 (24 hours after infusion), blood sampling and MR scanning with the same neuroimaging measurements were repeated. Treatments were crossed over with a washout period of approximately 18 to 21 days. Abbreviations: MRS, magnetic resonance spectroscopy; BDNF, Brain-derived neurotrophic factor.

## Supplementary Fig. S2

**Adjusted BDNF levels over time points and conditions.** Plasma BDNF concentrations were statistically adjusted for blood sampling time and log transformed. **(A)** Line plot illustrating mean adjusted plasma BDNF concentrations with error bars representing the standard error of the mean (SEM). Plasma BDNF levels in the ketamine condition showed a significant decrease from baseline to 24 hours post-infusion (t(25) = 2.39, α = 0.025, p = 0.0249), while no significant change was observed in the placebo condition (t(19) = 1.12, α = 0.025, p = 0.278). **(B)** Distribution of individual adjusted plasma BDNF concentrations at baseline and 24 hours post-infusion, stratified by treatment condition. Boxes represent median values with interquartile ranges; violin plots and jittered points indicate data distribution and individual data points, respectively. Abbreviations: BDNF, Brain-derived neurotrophic factor.

# Supplementary Figures

**Supplementary Fig. S1**


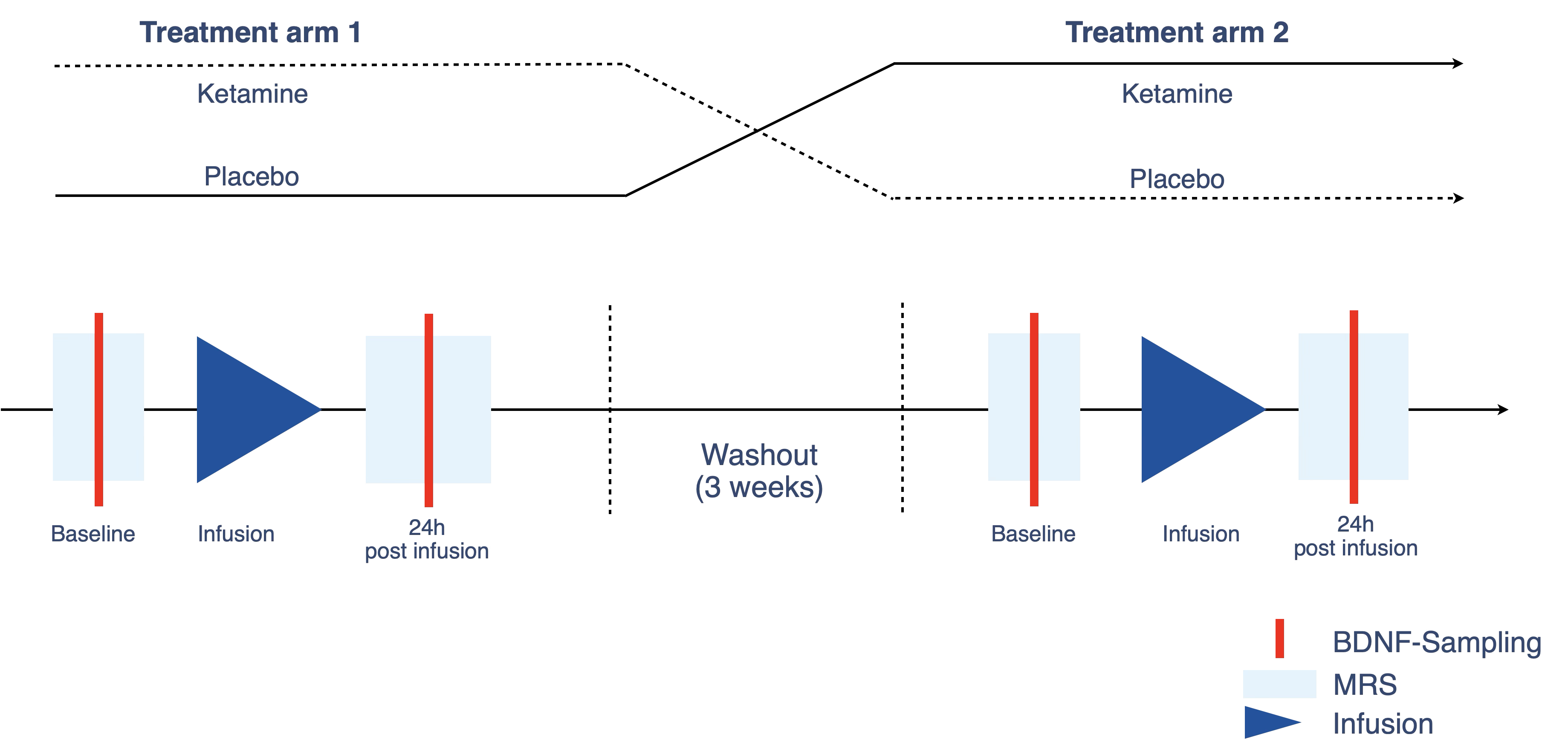


| A) | B) |
| --- | --- |
| 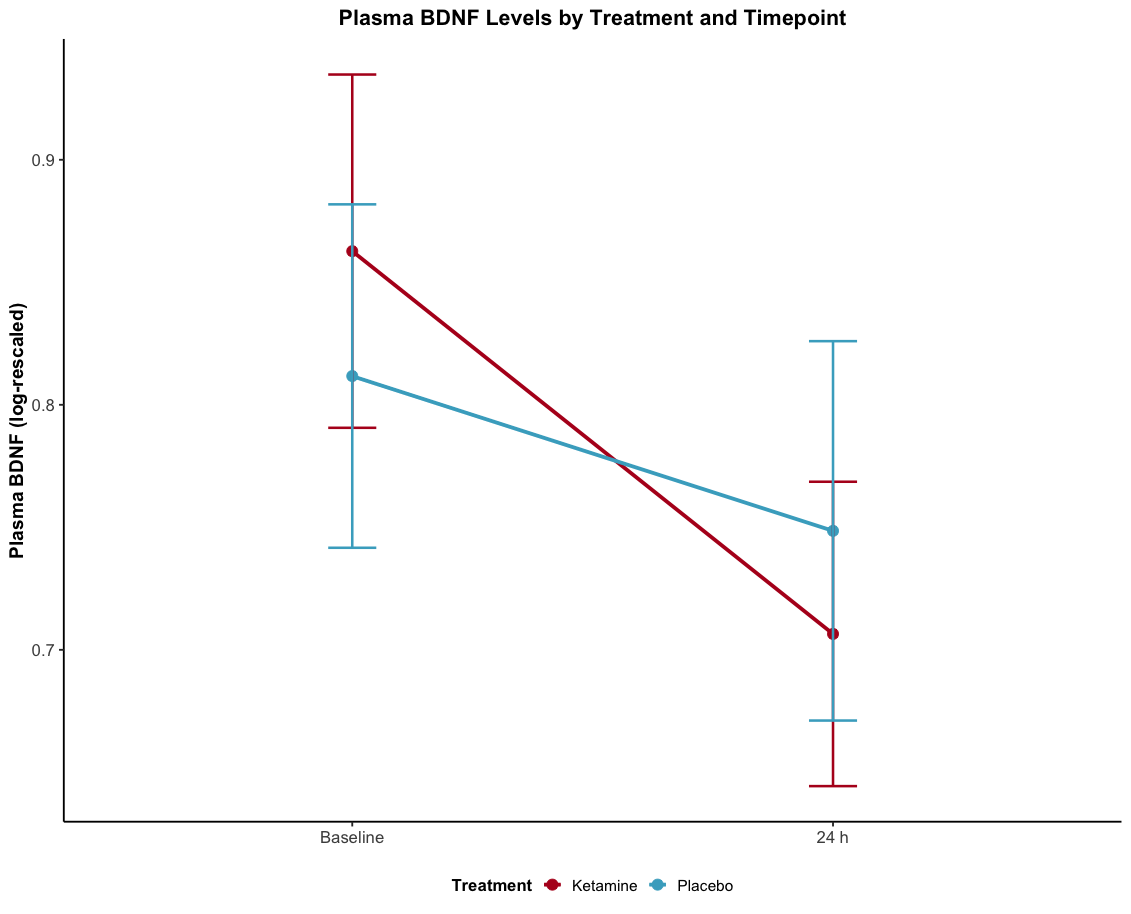 | 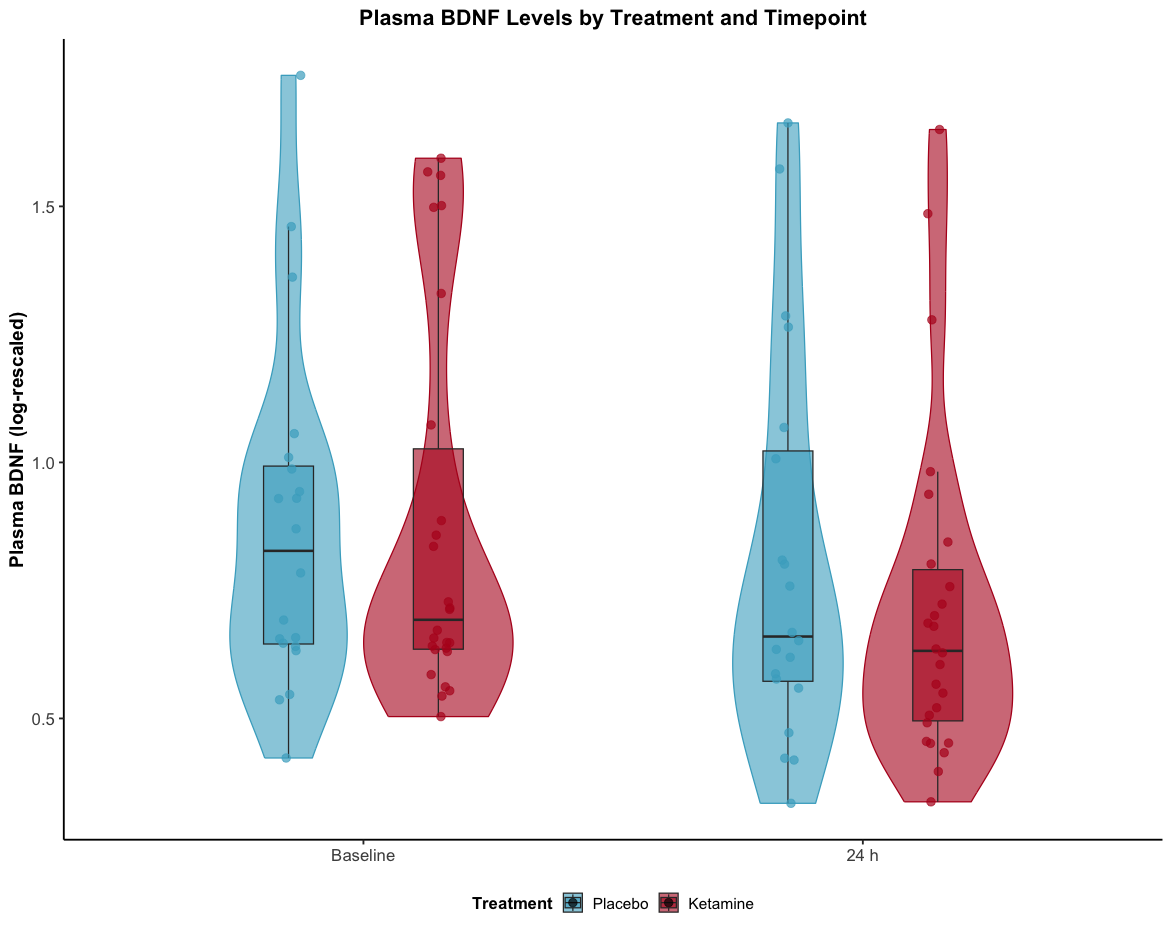 |

**Supplementary Fig. S2**

# Supplementary Tables

**Supplementary Table 1** Sample demographics and health variables

| Characteristic | Descriptive Statistic | Value |
| --- | --- | --- |
| Age (years) | mean [SD] | 25.1 [4.2] |
| BMI | mean [SD] | 25.0 [2.5] |
| V/V BDNF-Genotype | n [%] | 24 [68,6] |
| V/M BDNF-Genotype | n [%] | 9 [25,7] |
| M/M BDNF-Genotype | n [%] | 2 [5,7] |

Abbreviations: SD, Standard Deviation; BMI, Body Mass Index; V, Valine; M, Methionine; BDNF, Brain-derived neurotrophic factor
'*****' p < 0.05, '**.**' p < 0.1

**Supplementary Table 2** Results of the linear regression analysis on relative BDNF changes with predictors including treatment (S-Ketamine vs. placebo), relative Glu changes, their interaction, and age as covariates

| Predictor | ⁠*β* | SE | t-value | p-value |
| --- | --- | --- | --- | --- |
| (Intercept) | -0.044 | 0.253 | -0.174 | 0.862 |
| Relative Glu Change | -0.331 | 0.407 | -0.814 | 0.421 |
| Treatment (S-ketamine) | -0.15 | 0.086 | -1.75 | 0.088**.** |
| Age | -0.0001 | 0.009 | -0.014 | 0.989 |
| Relative Glu Change : Treatment (S-ketamine) | 1.006 | 0.48 | 2.1 | 0.042***** |

Abbreviations: Glu, Glutamate; SE, Standard Error

'*****' p < 0.05, '**.**' p < 0.1

**Supplementary Table 3** Results of the linear regression analysis on relative BDNF changes with predictors including treatment (S-Ketamine vs. placebo), relative Glu changes, their interaction, and BMI as covariates

| Predictor | ⁠*β* | SE | t-value | p-value |
| --- | --- | --- | --- | --- |
| (Intercept) | -0.051 | 0.4 | -0.128 | 0.899 |
| Relative Glu Change | -0.33 | 0.4 | -0.825 | 0.414 |
| Treatment (S-ketamine) | -0.15 | 0.086 | -1.749 | 0.088**.** |
| BMI | < 0.001 | 0.016 | 0.009 | 0.993 |
| Relative Glu Change : Treatment (S-ketamine) | 1.005 | 0.477 | 2.107 | 0.041***** |

Abbreviations: Glu, Glutamate; SE, Standard Error; BMI, Body Mass Index

'*****' p < 0.05, '**.**' p < 0.1

**Supplementary Table 4** Results of the linear regression analysis on relative BDNF changes with predictors including treatment (S-Ketamine vs. placebo), relative Glu changes, their interaction, and genotype (Val/Val, Val/Met) as covariates

| Predictor | ⁠*β* | SE | t-value | p-value |
| --- | --- | --- | --- | --- |
| (Intercept) | -0.032 | 0.068 | -0.465 | 0.645 |
| Relative Glu Change | -0.307 | 0.41 | -0.751 | 0.457 |
| Treatment (S-ketamine) | -0.152 | 0.087 | -1.754 | 0.087**.** |
| M/M Genotype | -0.022 | 0.168 | -0.131 | 0.897 |
| V/M Genotype | -0.063 | 0.096 | -0.659 | 0.514 |
| Relative Glu Change : Treatment (S-ketamine) | 1.004 | 0.479 | 2.094 | 0.043***** |

Note that the V/V genotype was selected as the reference category due to its larger sample size (n = 24) and is therefore represented implicitly in the model's intercept. Abbreviations: Glu, Glutamate; SE, Standard Error; V, Valine; M, Methionine

'*****' p < 0.05, '**.**' p < 0.1
